# Superior Analgesic Effect of an Active Distraction versus Pleasant Unfamiliar Sounds and Music: The Influence of Emotion and Cognitive Style

**DOI:** 10.1371/journal.pone.0029397

**Published:** 2012-01-05

**Authors:** Eduardo A. Garza Villarreal, Elvira Brattico, Lene Vase, Leif Østergaard, Peter Vuust

**Affiliations:** 1 Center of Functionally Integrative Neuroscience, University of Aarhus, Aarhus, Denmark; 2 The Royal Academy of Music, Aarhus and Aalborg, Denmark; 3 Department of Psychology, University of Aarhus, and Danish Pain Research Center, Aarhus University Hospital, Aarhus, Denmark; 4 Cognitive Brain Research Unit, Cognitive Science, Institute of Behavioral Science, University of Helsinki & Centre of Excellence for Interdisciplinary Music Research, University of Jyväskylä, Jyväskylä, Finland; 5 Department of Neuroradiology, Aarhus University Hospital, Aarhus, Denmark; University of Sydney, Australia

## Abstract

Listening to music has been found to reduce acute and chronic pain. The underlying mechanisms are poorly understood; however, emotion and cognitive mechanisms have been suggested to influence the analgesic effect of music. In this study we investigated the influence of familiarity, emotional and cognitive features, and cognitive style on music-induced analgesia. Forty-eight healthy participants were divided into three groups (empathizers, systemizers and balanced) and received acute pain induced by heat while listening to different sounds. Participants listened to unfamiliar Mozart music rated with high valence and low arousal, unfamiliar environmental sounds with similar valence and arousal as the music, an active distraction task (mental arithmetic) and a control, and rated the pain. Data showed that the active distraction led to significantly less pain than did the music or sounds. Both unfamiliar music and sounds reduced pain significantly when compared to the control condition; however, music was no more effective than sound to reduce pain. Furthermore, we found correlations between pain and emotion ratings. Finally, systemizers reported less pain during the mental arithmetic compared with the other two groups. These findings suggest that familiarity may be key in the influence of the cognitive and emotional mechanisms of music-induced analgesia, and that cognitive styles may influence pain perception.

## Introduction

The pain modulation system is influenced by several factors such as cognition and emotion, which can alter the perception of pain [1], [2]. Importantly, several studies have indicated that distraction from a nociceptive stimulus, or positive emotions elicited by an external stimulus, can reduce pain [3], [4], [5], [6], [7], [8]. It was recently discovered that distraction modulates pain differently from emotion [9], [10].

Music is an example of an external and distracting stimulus with cognitive and emotional features that can induce an analgesic effect [11], [12], [13], [14], [15], [16], [17]. Several studies indicate that music could play an important role as an adjunct treatment for medical disorders for different reasons: it has been found to reduce pain as well as the required dosage of analgesic medication necessary for treatment, and it is beneficial to an individual's overall well-being [17], [18], [19], [20], [21], [22], [23], [24]. However, there is still limited knowledge about which features of the music are responsible for the analgesic effect, and which neural mechanisms are involved, possibly due to the choice of poor control conditions or the lack of randomized controlled trials [25].

Recent studies have aimed to uncover the analgesic mechanisms of music using an experimental acute pain design. Mitchell, et al. 2006 [26] showed that music has a superior analgesic effect to an active distraction such as mental arithmetic. However, the music used in this study was self-chosen and familiar, and therefore, individual preferences and familiarity could enhance the drive to listen attentively to the music and thus act as a distractor from the pain. This was corroborated by work from the same group showing that familiar music increases pain tolerance more than unfamiliar music [27]. Roy et al. 2008 [28] showed that pleasant music reduces pain more than unpleasant music, and that the emotional valence is negatively correlated with the amount of pain reported. This is no surprise, since positive valence reduces pain regardless of the sensory system [26], [28], [29], [30]. However, the unpleasant music used in the Roy et al. study did not increase the pain as expected. Furthermore, even though this study used music unknown to the participants, the music could be considered mainstream and hence, possibly familiar to them. Therefore, the effect of familiarity may have a higher role in the mentioned studies.

Arousal is another emotional factor that has been related to pain relief and in music, arousal interacts with valence to reduce pain [29], [30], [31]. Therefore, valence and arousal are two interrelated emotional mechanisms that are clearly linked to the analgesic effect of music.

Lastly, an important factor that plays a role in the study of the analgesic effects of music is that the musical experience is highly individual. The individual variability in cognitive style has not yet been examined in earlier studies. Cognitive styles, such as being empathic and having a tendency to focus on emotions, or being systematic and having a tendency to focus on analytic structures, can affect the perception of an external stimulus by focusing attention to the different features and aspects of the stimulus [32], [33]. Because of this, individual cognitive style may contribute significantly to the variability of the analgesic effect of music. Understanding the features of the music that may reduce pain, and the internal mechanisms in the participant could minimize variability and increase the analgesic effect of music.

Evidence from most studies points to an analgesic effect of music, whereas other studies, particularly clinical ones, show no music-induced analgesic effect of music [34], [35]. This suggests that the analgesic effect of music is highly variable. The differences observed between studies could be explained by the variability of the musical features, the emotions involved, and the familiarity of the music used in the various studies.

In this study, we wanted investigate whether music reduces pain to a larger extent than an active distraction task when controlling for known analgesic mechanisms such as familiarity, valence, arousal, and individual cognitive style. To do so, we exposed healthy participants with different cognitive styles (empathizers, systemizers, balanced) [33] to experimental heat stimuli during four different listening conditions: Mozart music, environmental sounds, mental arithmetic and a control. The mental arithmetic was an active distraction task, whereas the rest were passive auditory stimuli. The Mozart music and environmental sounds were unfamiliar and matched for valence and arousal to study the attribution of these features to the analgesic effect. We hypothesized that the active and passive stimuli would have an analgesic effect when compared to the control, and that the environmental sounds and Mozart music would have a superior analgesic effect to mental arithmetic. We predicted that both environmental sounds and Mozart music would lead to similar pain ratings in participants if the main analgesic mechanisms were related to cognition and emotion, and not to the music itself. Finally, we hypothesized that valence, arousal, liking, and cognitive styles would influence the analgesic effect of the auditory stimuli. In particular, we expected that stimuli with positive valence, liked, and with low arousal would be the most effective in reducing pain perception. We further predicted that systemizer individuals would show stronger analgesic effects during more cognitively demanding and distracting tasks, whereas the pain perception in empathizers would be more affected by highly liked positive auditory stimuli.

## Materials and Methods

### Participants

Forty-eight native Danish speakers (24 male, 24 female), aged between 19 and 39 years (mean = 24, SD = 4), participated in the experiment. All participants were healthy, right handed, reported normal hearing and had minimal to no musical training. They had not consumed any analgesic medication in the 24 hours prior to the experiment. Participant recruitment was done via advertisements and a research recruitment website. Upon inclusion in the study, the participants filled out an online version of the Baron-Cohen Empathizer-Systemizer Quotients in Danish [33], [36]. Based on these results the participants were categorized and divided into three groups: Empathizers (8m/8f), Systemizers (8m/8f) and Balanced (8m/8f). Written informed consent was obtained from all participants and the study was conducted according to the Declaration of Helsinki. Participants received compensation for taking part in the experiment. Ethical permission was obtained from The Research Ethical Committee for Mid-Jutland Region, Denmark.

### Thermal stimuli and pain measures

The thermal stimuli were produced by a 3×3 cm contact thermode (Pathway model ATS from Medoc Ltd. Advanced Medical System, Israel) placed on the anterior surface of the forearms. The pain limits and threshold were investigated for each participant during calibration trials prior to the study in order to control for individual differences in pain perception. In accordance with Price et al. 1999 [37], we presented two trials with four different temperatures: 42, 43, 45, and 47°C in a random order. Each stimulus lasted ∼10 s and was separated by approximately 15–20 s. The participants rated pain intensity and unpleasantness on the Visual Analog Scale (VAS) (0–100 mm) at each temperature. An individual goal temperature was determined, which had to reflect pain ratings between 50–70 mm (moderate to high) in the VAS.

In the experiment, the individual goal temperature was used as the painful stimulus and was kept constant during the entire experiment to avoid high variability of the VAS scores between participants. To avoid habituation, the thermode was changed to a slightly different skin location on the forearm after every two experimental conditions. Both forearms were stimulated during the experiment. Each painful stimulus consisted of a plateau of 16 s with a rise/fall time of 2 s. The baseline temperature was 35°C. The thermal stimulus was rated using the VAS for pain intensity and unpleasantness. The scale ranged from “no pain” (left end of the scale) to “very intense” or “very unpleasant” (right end) (0–100 mm) [38].

### Auditory stimuli

Prior to the experiment, we conducted a pilot study intended for selecting musical pieces and environmental sounds most appropriate for this study. We recruited 18 healthy participants (9 males/9 females; mean age = 27) who listened to a pool of 16 environmental sounds and 19 musical excerpts. The environmental sounds were recordings from nature (edited from the sound effects library, Sound Ideas http://www.sound-ideas.com). There were four excerpts of each type: Fire, Water, Rain and Wind. The musical pieces were 19 different Mozart string compositions, virtually unknown to the layman. The participants were required to rate the stimuli according to valence (0 = unpleasant, 10 = pleasant), arousal (0 = environmental, 10 = stimulating), and liking (0 = not liked, 10 = liked). The results showed that Rain and Water for the environmental sounds and *“String Quartet No. 1 in G major, K. 80/73f (1770) – Adagio”* and *“Divertimento in E flat, K. 563 – Adagio”* for the music pieces, were rated as the most pleasant, liked and relaxing. In this pilot study, the participants also reported that pink noise was less distressing than white noise and therefore we choose to include the prior as a control.

In the experiment, we used these selected musical pieces and environmental sounds as well as the pink noise. Each auditory stimulus lasted 300 s (5 min) and we performed peak normalization on each of them. Peak normalization is an automated process in which the software scans the entire signal to find the loudest peak, and then adjusts each sample to a specific level. It is used to ensure that the signal peaks at the loudest level allowed in a digital system and does not cause clipping in the sound system. After the experiment, the participants were asked if they had previously listened to the musical piece, as familiarity with the music would influence the analgesic effect.

The PASAT (Paced Auditory Serial Addition Test) was chosen as the active distraction task. For details about the PASAT see Gronwall, 1977 [39]. The PASAT consisted of a woman's voice in Danish dictating numbers every three seconds. The task for the participant consisted of adding together two of the numbers at a time, the last dictated plus the new dictated number.

**Figure 1:**
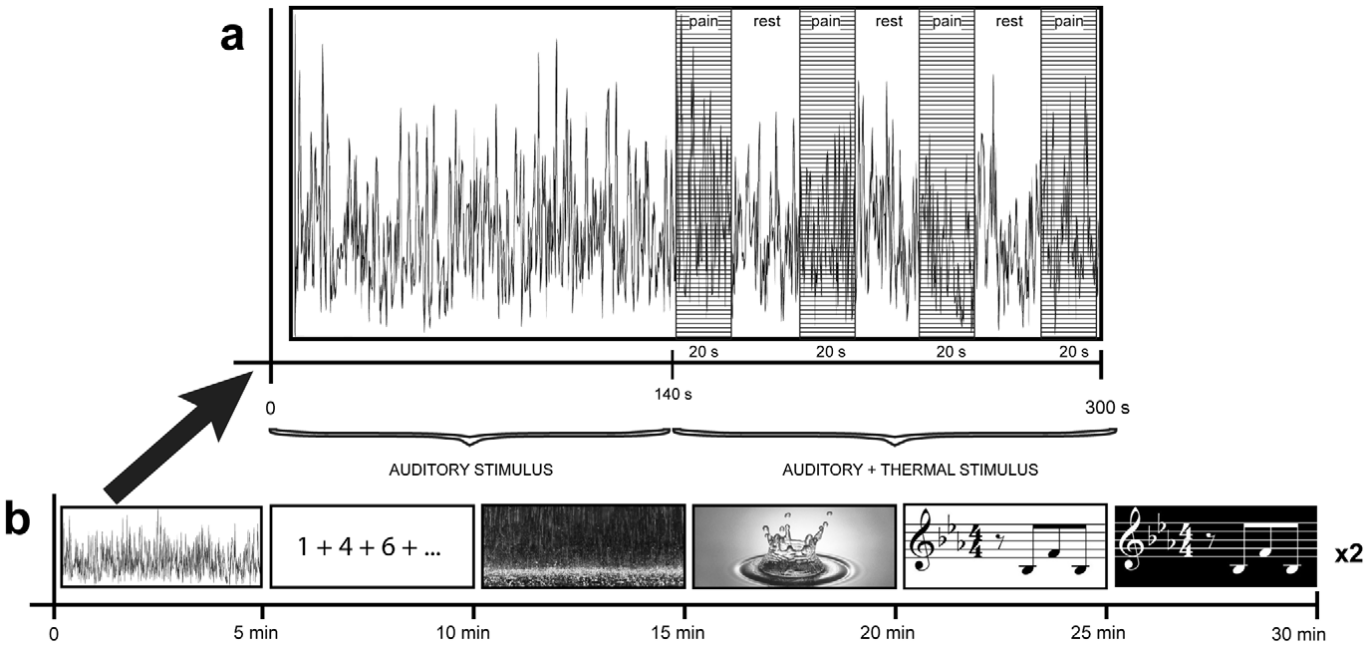
Paradigm. The complete paradigm lasted approx. 60 min. **a.** Here we show an example of the structure of each condition. The first 140 s consisted of only the passive listening of the auditory stimulus (i.e. noise). Afterwards, the four thermal (pain) stimuli were delivered, with the auditory stimulus still playing. “Pain” refers to when the thermal stimulus was ON, and “rest” refers to when it was OFF (no pain, baseline). The participants rated the pain during “rest”. **b.** Here we show the structure of a complete run. It consisted of the five random conditions (noise, rain, water, music1, music2), lasting 5 min each, for a total of 30 min for one run. The whole paradigm consisted of two runs (60 min).

Each experimental condition lasted 300 s (5 min). The first 140 s consisted of passive listening (or active if it was the PASAT) of the auditory stimulus, whereas the last 160 s consisted of the auditory stimulus plus four thermal stimuli (Fig. 1). Listening to music and other pleasant auditory stimuli elicits emotional responses that might not be immediate, and such responses are thought to be important for the analgesic effect of music and reduction of anxiety [11]. Therefore, the passive listening period was included to ensure that the emotions and mood induced by each auditory stimulus were present as much as possible. The PASAT condition was considered the “active condition”, and the Noise, Rain, Water, Music 1 and Music 2 were considered the “passive conditions”.

### Emotional measures

After the experiment, the participants rated the auditory stimuli on a 10-point Likert scales for valence (0 = unpleasant, 10 = pleasant), liking (0 = does not like, 10 = likes), and arousal (0 = relaxing, 10 = stimulating).

### Cognitive styles

The participants answered the Baron-Cohen Empathizer Systemizer Quotients (two questionnaires) [33], [36], which can divide the population into three groups: Empathizers (more empathic and social), Systemizers (attracted to patterns in objects and events) and Balanced (in between). Empathizers are may be attracted to the emotional content of the music, whereas the Systemizers may be attracted to musicianship and performance level [32]. Although the Baron-Cohen quotient has not been used in pain studies, it has been related to music listening styles [32]. Thus, the auditory stimuli may influence cognitive style and potentially influencing cognitive and emotional mechanisms that reduce pain.

The categorization of the groups was done using the points from each questionnaire that were then processed using the method described in Wheelwright, et al. 2006 [40]. In short, we used these formulas: *S = (SQ – 55.6)/150* and *E = (EQ – 44.3)/80*, then *D = (S – E)/2*, where SQ (Systemizer quotient) and EQ (Empathizer quotient) are the points from each of the questionnaires. The resulting *D* was then used to find the category using the following axioms: *If D<−.21, then ‘Extreme Empathizer’ (EE); if D≥−.21 but <−.041, then ‘Empathizer’ (E); if D≥−.041 but <.040, then ‘Balanced’ (B); if D≥.040 but <.21, then ‘Systemizer’ (S); if D>.21, then ‘Extreme Systemizer’ (ES)*. For the purpose of this study, EE was merged with E into the ‘Empathizer’ category, and ES was merged with S in the same fashion. The mean points obtained in each questionnaire by each group are shown in Table 1.

**Table 1:** Baron-Cohen E/S Quotient scores.

	EQ		SQ	
	*Mean*	*SD*	*Mean*	*SD*
Empathizers	57.13	6.94	51.25	12.57
Systemizers	39.75	6.33	71.75	11.97
Balanced	49.06	8.71	61.13	13.89

EQ = Empathizer Quotient, SQ = Systemizer Quotient, SD = Standard deviation.

### Procedure

The participants were contacted prior to the experiment in order to get the information regarding the thermal stimulation and they were asked to answer the Baron-Cohen questionnaire. Once the cognitive style was determined, each participant was assigned to a group until the quota was fulfilled. The participants were asked to come to the laboratory and were told that during the study they were going to receive painful stimuli while listening to different types of auditory stimuli. When entering the laboratory they were told that their task was to rate the pain. The experiment took place in a sound proof white room without windows. Instructions for all participants were identical and given by the same male experimenter, who was the only person present during the experiment. The participants were trained to use the VAS and were familiarized with the thermal stimuli by investigating pain limits and threshold. They were also trained to perform the PASAT. They were seated comfortably in a chair in front of a monitor and were given a mouse to rate pain using a computerized VAS. To minimize confounds, a panel wall stood between the experimenter and the participant to avoid visual contact, and the participants were told that the experimenter could not see their pain scores once the experiment started. The auditory stimuli were presented using headphones (Philips Hi Fi Stereo headphones® SH P8900) at an individual comfortable sound intensity level that remained constant throughout the experiment. The auditory and thermal stimuli were presented and controlled by a computer using Presentation® software (Version 14.0, www.neurobs.com). The individual goal temperature was kept constant during the study.

The paradigm included six conditions: two musical excerpts (Music1, Music2), two environmental sounds (Rain, Water), an active distraction task (PASAT), and a control (Noise). Each condition lasted 300 seconds (5 minutes) for a total time of 30 minutes per run. Each experiment consisted of two runs per participant with one minute of rest in between (Fig. 1). During each condition, the auditory stimuli were presented for the entire 300 s. During the first 140 s, the participants listened passively (or actively for the PASAT) to the auditory stimulus. During the following 160 s, they also received four consecutive painful thermal stimuli. After each painful stimulus, the participants had 20 seconds to rate it for intensity and unpleasantness. The conditions were quasi-randomized, making sure the two environmental sounds (Rain/Water) and the music pieces (Music1/Music2) did not follow each other. After the experiment, the participants rated the auditory stimuli for valence, liking and arousal, and reported that the music pieces were unfamiliar to them.

### Statistical analysis

The statistical analysis was performed using SPSS version 17.0 (SPSS Inc., Chicago IL). First, we compared the emotional measures (valence and arousal) between groups in the pilot study vs. in the experiment, with *a one-way* ANOVA to determine if the overall ratings were similar. Then we compared each of the conditions (Rain, Water, Music1, Music2) using a *t-test* as a *post hoc* analysis. This was to confirm that the auditory stimuli used in the experiment evoked the expected emotions. As the main analysis of the experiment, we compared the pain ratings between conditions using *repeated-measures* ANOVA. The dependent variables were pain intensity (PI) and pain unpleasantness (PU). Furthermore, we analyzed the emotional ratings between conditions again using *repeated-measures* ANOVA. The dependent variables were valence, arousal and liking.

For both *repeated-measures* ANOVAs (pain and emotion) we studied the six-level within-subjects factor “condition”: Noise, PASAT, Rain, Water, Music1, Music2, and the between-subjects factor “cognitive style”. We performed single *pre-hoc* contrasts using Noise as the contrasting condition, as well as *post-hoc* pairwise comparisons to investigate differences between the all conditions. The Bonferroni correction was used to control for multiple comparisons. Type I errors were controlled for by using Mauchly's test and the Greenhouse–Geisser epsilon when appropriate.

Finally, we performed a Pearson correlation analysis to determine the relationship between pain and emotion ratings. For this, we computed an index of analgesia for each condition by subtracting the pain ratings of a given condition from the ratings during the control (Noise) condition. These subtracted pain scores (PIs, PUs) were analyzed with valence, liking and arousal. Because of previous knowledge regarding correlations between pain and emotion, the analysis was one-tailed. The alpha level for all statistical analyses was .05, unless stated otherwise. The effect sizes of the main analyses were calculated using partial eta squared (η^2^_p_). Effect sizes of the contrasts were calculated using the following formula:
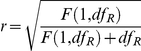



## Results

### Pilot study

The *one-way* ANOVA (Valence: F (1, 248) = .865, p = .35; Arousal: F (1, 248) = 3.34, p = .07) and the individual *t-tests* showed no significant differences between the pilot study ratings and those of the experiment. This indicates that Rain, Water, Music1 and Music2 were rated similarly in the pilot study and the experiment; hence the stimuli evoked the intended emotions with respect to valence, liking and arousal.

### Analysis of pain

**Figure 2:**
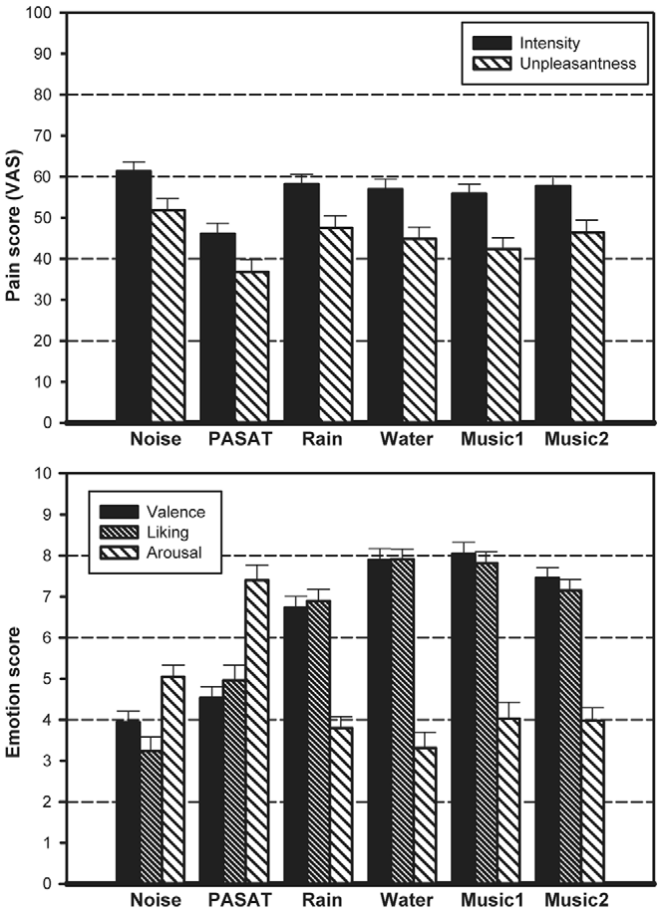
Pain and emotion. **a.** Mean values of the VAS in each condition. 0 = “no pain” and 100 = “worst pain”. **b.** Mean values of the ratings of valence, liking and arousal in each condition. Valence (0 = “unpleasant”, 10 = “very pleasant”), liking (0 = “doesn't like”, 10 = “likes”), arousal (0 = “relaxing”, 10 = “stimulating”).

Figure 2 shows the descriptive statistics of pain and emotion ratings for all the conditions. The *repeated-measures* ANOVA showed a significant within-subjects effect of condition (Table 2) for PI and PU, suggesting that the conditions were rated differently. The contrasts showed that Noise was rated to be significantly more painful than the rest of the conditions, which was expected as Noise is the control condition. The effect sizes revealed that the PASAT had the highest effect in both pain dimensions, and Rain had the lowest. The *post-hoc* pairwise comparisons showed the PASAT had significantly lower PI ratings than Rain, Water, Music1 and Music2. On the other hand, in the PU the PASAT was not significantly different from Music1, suggesting that Music1 reduced PU to the same extent as the PASAT. In PI, the passive conditions (Rain, Water, Music1 and Music2) were not significantly different from each other, suggesting that PI was similarly rated across conditions. In PU, the passive conditions were also not significantly different from each other, except Rain, which was significantly more painful than Music1.

**Table 2:** Results of the repeated-measures ANOVA of the pain ratings.

	PI	PU
***Within-subjects***		
Condition	F (3.28, 147.36) = 22.58, p = .000, η^2^_p_ = .33	F (3.35, 150.92) = 12.56, p = .000, η^2^_p_ = .22
***Contrasts***		
Noise vs. PASAT	F (1, 45) = 50.22, p = .000, r = .73	F (1, 45) = 30.12, p = .000, r = .63
Noise vs. Rain	F (1, 45) = 4.70, p = .036, r = .30	F (1, 45) = 4.26, p = .045, r = .29
Noise vs. Water	F (1, 45) = 15.32, p = .000, r = .50	F (1, 45) = 18.55, p = .000, r = .54
Noise vs. Music1	F (1, 45) = 13.01, p = .001, r = .47	F (1, 45) = 22.31, p = .000, r = .58
Noise vs. Music2	F (1, 45) = 6.49, p = .014, r = .36	F (1, 45) = 9.19, p = .004, r = .41
***Pairwise comparisons***		
PASAT vs. Rain	p = .000	p = .002
PASAT vs. Water	p = .000	p = .013
PASAT vs. Music1	p = .000	n.s.
PASAT vs. Music2	p = .000	p = .013
Rain vs. Water	n.s.	n.s.
Rain vs. Music1	n.s.	p = .035
Rain vs. Music2	n.s.	n.s.
Water vs. Music1	n.s.	n.s.
Water vs. Music2	n.s.	n.s.
Music1 vs. Music2	n.s.	p = .049
***Between-subjects***		
Cognitive type	n.s.	n.s.
**Interaction** = Condition×Cognitive type	F (10, 225) = 2.04, p = .05	n.s.

n.s. = Not significant, PI = Pain intensity, PU = Pain unpleasantness, r = effect size, η^2^_p_ = effect size partial eta squared.

**Figure 3:**
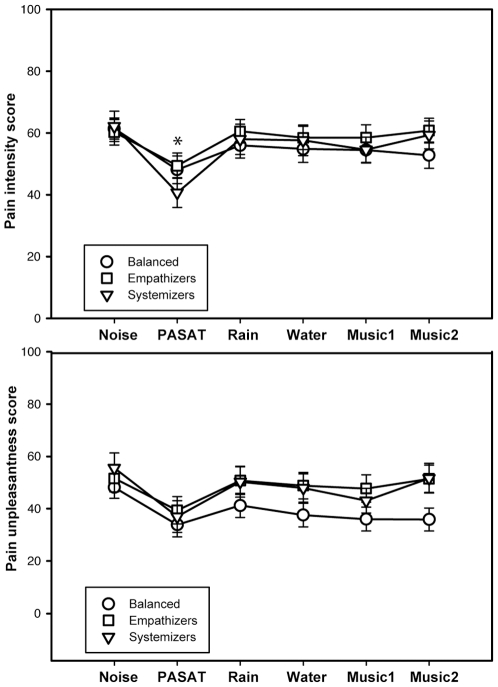
Cognitive styles. **Top.** Mean pain intensity scores (VAS) for each cognitive style and condition. The * indicates statistical significance. **Bottom.** Mean pain unpleasantness scores (VAS) for each cognitive style and condition.

The between-subjects effect of cognitive style in PI and PU was not significant, suggesting that empathizers, systemizers and balanced rated PI and PU similarly. However, there was a significant interaction between condition and cognitive type in PI, due to the systemizers reporting less PI during the PASAT condition than the empathizers and balanced participants (Fig. 3).

### Analysis of emotion

The *repeated-measures* ANOVA showed a significant within-subjects effect of condition (Table 3) for valence, liking and arousal, indicating that the conditions were rated differently. The contrasts showed that Noise was rated significantly different than the rest of the conditions in valence (least pleasant), liking (least liked) and arousal (more stimulating), except that Noise and PASAT conditions were rated similarly for valence. The *post hoc* pairwise comparisons showed that the PASAT was significantly different than Rain, Water, Music1 and Music2 in valence (less pleasant), liking (less liked) and arousal (more arousing). In liking and arousal, the passive listening conditions did not differ significantly, suggesting they were rated similarly. In valence, only Rain was significantly less pleasant than Water and Music1. The rest of the conditions in valence were not significantly different.

**Table 3:** Results of the repeated-measures ANOVA of the emotion ratings.

	Valence	Liking	Arousal
***Within-subjects***			
Condition	F (3.82, 171.85) = 50.06, p = .000, η^2^_p_ = .53	F (3.73, 175.28) = 37.70, p = .000, η^2^_p_ = .45	F (2.80, 125.75) = 22.93, p = .001, η^2^_p_ = .34
***Contrasts***			
Noise vs. PASAT	n.s.	F (1, 45) = 13.35, p = .001, r = .48	F (1, 45) = 28.72, p = .000, r = .62
Noise vs. Rain	F (1, 45) = 46.71, p = .000, r = .71	F (1, 45) = 60.34, p = .000, r = .76	F (1, 45) = 8.30, p = .006, r = .39
Noise vs. Water	F (1, 45) = 88.05, p = .000, r = .81	F (1, 45) = 91.74, p = .000, r = .82	F (1, 45) = 13.20, p = .001, r = .48
Noise vs. Music1	F (1, 45) = 100.73, p = .000, r = .83	F (1, 45) = 97.58, p = .000, r = .83	F (1, 45) = 3.58, p = .065, r = .27
Noise vs. Music2	F (1, 45) = 87.31, p = .000, r = .81	F (1, 45) = 85.31, p = .000, r = .81	F (1, 45) = 5.19, p = .027, r = .32
***Pairwise comparisons***			
PASAT vs. Rain	p = .000	p = .005	p = .000
PASAT vs. Water	p = .000	p = .000	p = .000
PASAT vs. Music1	p = .000	p = .000	p = .000
PASAT vs. Music2	p = .000	p = .001	p = .000
Rain vs. Water	p = .013	n.s.	n.s.
Rain vs. Music1	p = .001	n.s.	n.s.
Rain vs. Music2	n.s.	n.s.	n.s.
Water vs. Music1	n.s.	n.s.	n.s.
Water vs. Music2	n.s.	n.s.	n.s.
Music1 vs. Music2	n.s.	n.s.	n.s.
***Between-subjects***			
Cognitive type	n.s.	n.s.	n.s.

n.s. = Not significant, r = effect size, η^2^_p_ = effect size partial eta squared.

The between-subjects effect of cognitive style was not significant for valence, liking and arousal; meaning Empathizers, Systemizers and Balanced rated the emotions similarly.

### Correlation analysis

There was a significant low negative correlation between PIs and valence (p = .006, r = −.16), and a significant medium positive correlation between PIs and arousal (p<.001, r = .26). Therefore, the more pleasant and relaxing the auditory stimulus was, the less pain intensity was perceived. There was near significant low correlation with liking (p = .057, r = −.10). For PUs, there was a significant positive correlation with arousal (p<.005, r = .18), but not with valence (p = .08, r = −.09) or liking (p = .41, r = −.02). Thus the more relaxing the auditory stimulus was, the less pain unpleasantness was perceived.

## Discussion

In this study we found that the active distraction, represented by mental arithmetic, reduced pain more than the passive distractions, which included music and sounds. Environmental sounds and Mozart music had an analgesic effect, however they reduced pain similarly, which is probably explained by their matched valence, liking and arousal as rated by the participants. Pain intensity was significantly correlated with valence and arousal, whereas pain unpleasantness was only correlated with arousal. Finally, participants with the cognitive style systemizer perceived less pain intensity than empathizers and balanced during the mental arithmetic condition.

### Distraction vs. music

Contrary to our hypothesis, the active distraction by PASAT reduced pain intensity more than the music and environmental sounds. Also, PASAT reduced pain unpleasantness more than Rain, Water, Music2, but not Music1. Thus, it is clear that the active distraction was superior to the passive distractions in reducing pain in general.

The analgesic effect of the PASAT can be considered to reflect distraction as its mechanism [10], [41]. Another analgesic mechanism involved in the pain relieving effect of the PASAT may be stress-induced analgesia (SIA), where exposure to a stressful stimulus suppresses pain [42]. Performing mental arithmetic while receiving and rating pain may provide enough stress to elicit this survival mechanism. Our results are in contrast with the findings of Mitchel et al. 2006, which showed that music was superior to PASAT in relieving pain. However, several differences in experimental design may explain this discrepancy. Mitchell and colleagues elicited pain using the cold pressor, a technique that is thought to emulate chronic pain, whereas we used localized heat eliciting acute pain [43]. The different types of experimentally elicited pain could be affected differently by stimuli such as music. Moreover, although they measured both pain tolerance and intensity, they only found a difference in pain tolerance and not in pain intensity. In contrast, we did find a difference in pain intensity and unpleasantness. Most importantly, in the study by Mitchell et al. the music was self-chosen and familiar, whereas in our study the music was experimenter-chosen and unfamiliar. Mitchel et al. showed that familiar music provides a higher pain tolerance than unfamiliar music [26]. Therefore, familiarity with the music may be crucial to direct the attention to the music, increasing the distraction from the noxious stimulus.

### Music vs. sounds

The environmental sounds and Mozart music both reduced pain significantly compared to the noise. This provides further evidence for the analgesic effect of music, and also for the analgesic effect of auditory stimuli in general. The environmental sounds and Mozart music were unfamiliar to the participants and were characterized by a comparable range of valence (high), liking (high) and arousal (low). Both environmental sounds and Mozart music reduced the same amount of pain intensity (the sensory perception of the noxious stimulus). Also, the sounds and music had similar ratings of arousal. On the other hand, the condition Rain was associated with the highest ratings of pain unpleasantness (the emotional perception of the noxious stimulus), whereas Music1 had the lowest rating when compared to noise (and significantly differed from Rain). Rain was also the condition with the lowest valence and liking, whereas Music1 had the highest. Moreover, Rain was significantly different than Water and Music1 in valence. In sum, our results suggest that the analgesic effect of music is probably not due to features of the music but more to cognitive and emotional factors, as we showed that music had similar analgesic effects to environmental sounds when valence, liking and arousal ratings were similar.

The correlation analysis shows a negative relationship between valence and pain intensity, and a positive relationship between arousal and pain intensity. Although the size of the correlation coefficients is small, it supports the results from Roy et al. 2008, in which they showed that valence and arousal correlate with pain intensity and unpleasantness. In relation to pain unpleasantness, we only found correlation to arousal, but not to valence. All of our auditory stimuli were unfamiliar and experimenter-chosen, which can explain the low or lack of correlations. Familiarity is a long-term recognition memory process that refers to a subjective state of awareness according to prior experience [44]. This memory process is related to hedonistic judgments such as listening to preferred music [45]. Recent studies show low or lack of correlations of valence and arousal with unfamiliar music, probably due to lack of emotional engagement [46], [47]. Thus, familiarity could be key in the induction of analgesic effects related to emotional and reward mechanisms by means of memory and prior exposure. Moreover, this effect may also be due to perceived control, a known cognitive analgesic mechanism [2], [48]. Therefore, even though the sounds and the music in our study reduced pain, as the participants were not emotionally entangled with the auditory stimuli, the analgesic mechanisms might be less related to emotion than previously thought when the music is not familiar.

### Cognitive style

Sytemizers perceived less pain during the mental arithmetic than empathizers and balanced participants (Fig. 3). Systemizing is the drive to analyze variables, derive the underlying rules that govern the behavior of a system, and to control and construct them [33], [36], [49]. Because of this, they may be attracted to patterns and complex stimuli. Thus, systemizers may find the mental arithmetic more entraining and distracting than the passive distractions. This could explain the reduction in their pain perception in the PASAT condition. In contrast, empathizers and balanced cognitive styles were not related to increased analgesic effects in any condition. Empathizing is the drive to identify another person's emotions and thoughts to respond to these with an appropriate emotion, to predict behavior and to care about the feelings of others. Balanced refers to the participants with similar systemizing and empathizing scores. These two cognitive styles were not related to responses to auditory stimuli that may influence pain perception. There are several possible explanations for this: 1) Cognitive styles may not influence emotional and cognitive mechanisms of passive auditory perception, 2) the Baron-Cohen E-S Quotient may not reveal emotional mechanisms and responses, 3) the main analgesic mechanisms of auditory stimuli are not emotional but cognitive when the stimuli are unfamiliar. Future studies should investigate which of these explanations are most likely responsible for the effect. Overall, our study is the first to suggest and show that cognitive styles, particularly systemizing and empathizing quotients, may affect pain perception. Further research in systemizers could study other stimuli or music with complex features that may be more distracting to them.

### Implications and future directions

In summary, we found significant effects of a primary task on pain perception. In particular, a task involving active distraction was superior to unfamiliar passive tasks to reduce pain. The Mozart music reduced pain as well, however it had the same effect as environmental sounds with similar ratings of valence, arousal and liking. This suggests that it is valence, arousal and liking that seem to drive the analgesic effect of music rather than the music itself. Familiarity with the music may influence the emotional mechanisms to modulate the pain. When the music is unfamiliar, the main analgesic mechanisms may be instead cognitive. The results also show that the cognitive systemizing style influenced the analgesic effect of the active distraction only. Nevertheless, considering its significant analgesic effect compared to noise (although smaller than the PASAT) in the clinical context, music used as an analgesic adjuvant, would be preferable to mental arithmetic as the PASAT could be highly arousing and stressful for the patient. Furthermore, the PASAT task is highly dependent on individual cognitive abilities and mental state and may not be feasible in certain patient whereas listening to music is affordable and pleasant to almost everybody. Future studies should use neuroimaging methods, such as fMRI, to further understand the neural mechanisms behind the analgesic effects of music and environmental sound listening in acute and chronic pain.
